# Application of Massive Open Online Course to Grammar Teaching for English Majors Based on Deep Learning

**DOI:** 10.3389/fpsyg.2021.755043

**Published:** 2022-01-07

**Authors:** Minghui Du, Yiqun Qian

**Affiliations:** ^1^School of Foreign Language Education, Nanjing University of Chinese Medicine, Nanjing, China; ^2^School of Foreign Languages, Changzhou University, Changzhou, China

**Keywords:** MOOC, grammar teaching methods for English majors, deep learning, teaching application, self-reflection and self-criticism

## Abstract

The study aims to explore the roles of Massive Open Online Courses (MOOCs) based on deep learning in college students’ English grammar teaching. The data are collected using a survey. After the experimental data are analyzed, it is found that students have a low sense of happiness and satisfaction and are unwilling to practice oral English and learn language points in English learning. They think that college English learning only meets the needs of CET-4 and CET-6 and does not take it as the ultimate learning goal. After the necessity and problems in English grammar teaching are discussed, the advantages of flipped classrooms of MOOCs are discussed in English grammar teaching. A teaching platform is constructed to study the foreign language teaching mode under MOOCs, and classroom teaching is combined with the advantages of MOOCs following the principle of “teaching students according to their personalities” to improve the listening, speaking, reading, writing, and translation skills of foreign language majors. The results show that high-quality online teaching resources and the deep learning-based teaching environment can provide a variety of interactive tools, by which students can communicate with their peers and teachers online. Sharing open online communication, classroom discussion, and situational simulation can enhance teachers’ deep learning ability, like the ability to communication and transfer thoughts. Constructivism with interaction as the core can help students grasp new knowledge easily. Extensive communication and interaction are important ways for learning and thinking. The new model provides students with profound learning experience, expands the teaching resources of MOOCs around the world, and maximizes the interaction between online and offline teachers and students, making knowledge widely rooted in the campus and realizing the combination of online resources and campus classroom teaching. Students can learn the knowledge through autonomous learning and discussion before class, which greatly broadens the learning time and space. In the classroom and after class, the internalization and sublimation of knowledge are completed through group cooperation, inquiry learning, scenario simulation, display, and evaluation, promoting students to know about new knowledge and highlighting the dominant position of students.

## Introduction

Recently, the rise of Massive Open Online Courses (MOOCs) in China attracts the attention of the domestic educational circles. Some domestic colleges and universities cooperate with Netease and Aike, the two major online education companies, to develop new online courses, and others start to follow suit (Baudewyns et al., 2016). With the help of the Internet, the roles of MOOCs are summarized as follows: their learning resources are extremely rich; the videos are simple and intuitive; the machine evaluation system can score useful information fast and respond timely (Hampton et al., 2017). Therefore, it can be said that the arrival of MOOCs brings many new opportunities and ideas to learners, and lays the foundation for online education (Chen, 2019). Grammar is very important and is the foundation of a language. As China is not an English-speaking country, learning English here becomes relatively difficult (Goussakova, 2016). Although classroom English teaching seems to be improved in recent years, students’ understanding ability and transformation efficiency are still not satisfactory (Hudson et al., 2018). The most important reason is that ordinary classrooms cannot provide English learning resources for students, and the key language points are not systematic (Sunar et al., 2017). MOOCs come into being to solve the problem encountered in English teaching, and their teaching modes are different from those of traditional teaching modes, and their knowledge systems are complete and logical so that students can understand and English grammar more clearly and help them build the corresponding grammar structure in their knowledge reserves, making English learning more efficient and improving students’ confidence in English learning and communication (Kellogg et al., 2013). In addition, MOOCs subdivide English knowledge and combine it with a video to explain the language point. A 10-min video can make the student understand the language point more efficiently and profoundly (Kastrati et al., 2019). The online teaching method combined with the traditional teaching method is more scientific so that students are more likely to grasp knowledge (Sarah, 2019).

The current teaching philosophy of MOOCs is to put learners at the center and vigorously stimulate their interest and willingness to learn. MOOCs are open on an online platform and provide students with rich learning resources and materials. Peers who have common interests can make learning plans and personalized programs together, which promotes their knowledge learning and interest development (Teeple et al., 2020). This open platform also displays students’ performance so that teachers can change their teaching plans according to students’ performance. MOOCs break the routine of the traditional teaching mode, stimulate students’ enthusiasm for learning English grammar, and develop their divergent thinking to learn by themselves. Their ideas and questions are uploaded on the network platform and answered immediately, improving their learning efficiency (Lan et al., 2019). The platform focuses on the application of information technology to English grammar teaching. Teachers should change their teaching concepts and teaching modes, so that the teaching process is optimized and the teaching quality is improved.

Based on the deep learning theory of MOOCs, the corresponding MOOCs’ teaching platform is constructed to study the foreign language teaching mode under MOOCs, and classroom teaching is combined with the advantages of MOOCs strictly following the principle of “teaching students according to their ability” to improve foreign language students’ listening, speaking, reading, writing, and translation skills. Through the online and offline foreign language teaching mode, students’ desire for foreign language learning is fully stimulated, the classroom completion rate and academic performance are improved, and the problems in learning foreign language under single MOOCs mode and the 3P classroom teaching mode are solved. The research results show that the high-quality online teaching resources and teaching environment based on deep learning can provide a variety of interactive tools, by which students can communicate with peers and teachers online, realize the deep combination of online resources and campus classroom teaching, and maximize the interaction between online and offline teachers and students.

## Introduction of Massive Open Online Courses in English Grammar Teaching

### Change of the Teaching Mode of English Grammar Based on Massive Open Online Courses

Massive Open Online Courses about English grammar teaching are divided into three modes, which are the online and offline auxiliary mode, the online and offline mixed teaching mode, and the online and offline integration mode (Lin et al., 2017). The online and offline integration teaching mode is that teachers obtain the desired teaching resources from MOOCs and select the appropriate content for offline teaching after the resources are sorted out. MOOCs include a lot of high-quality teaching courses and courseware (Li and Xing, 2021). This shows that MOOCs and English grammar teaching are well integrated (LEI, 2016). Nowadays, grammar courses are carried out by using many advanced teaching ideas and scientific teaching methods from MOOCs, and some great breakthroughs have been made (Mubarak et al., 2020).

The online–offline hybrid mode requires the teacher to focus on the traditional offline teaching concepts and methods, to divide the content of MOOCs into fragments, and to insert them into the corresponding teaching procedures, making the curriculum more diversified. However, grammar teaching should not be disconnected from the tradition (Qian et al., 2018) because it is not enough to use only the knowledge fragments in MOOCs and the traditional teaching method should also be used to transfer each language point to English grammar class (Rogoza et al., 2018). In addition, some senior teachers have their own teaching modes. Teachers can integrate other modes on the MOOC platform with the advantages of different methods to enrich their experience and evoke the enthusiasm of students in the classroom learning (Sun et al., 2018).

The online and offline auxiliary mode is a teaching method with a high degree of freedom (Wu and Song, 2019). Teachers find useful learning resources in MOOCs and recommend them to students, and students can choose the more understandable methods according to their learning progress and willingness, avoiding the phenomenon of passive learning. The essence of this method has nothing to do with traditional offline teaching (Wu et al., 2019). It is only used to arouse students’ interests in learning English, so that the transmission of knowledge is not limited to the classroom (Wu et al., 2020). Students can acquire the knowledge that they are interested in MOOCs to enrich their knowledge and broaden their horizons. MOOCs can also evaluate students’ performance, for example, some special tests are used to help students better understand their learning states and test their learning outcomes.

The abovementioned three modes are all the teaching modes in MOOCs. The auxiliary mode and the hybrid mode are based on English teaching (Wu and Wu, 2017). The integration of MOOCs and offline courses mainly depends on the development speed and integrity of MOOCs. MOOCs can overcome the disadvantages of traditional courses, and are very popular on the platform (Weng, 2018). The size of online education in China is also growing, as shown in Table 1.

**TABLE 1:** Size of online education in China from 2012 to 2020.

Year	2012	2013	2014	2015	2016	2017	2018	2019	2020
User scale (10,000 persons)	4,228	5091.4	6,174	7320.9	8857.7	10979.9	13539.8	16401.1	19485.3

### Integration of Massive Open Online Courses and English Grammar Teaching

**FIGURE 1:**
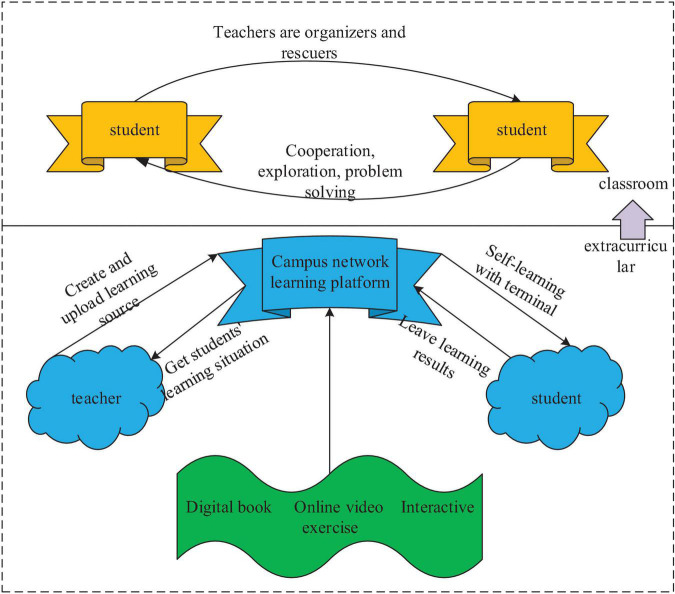
Practice of the flipped classroom.

Frequent communication between students and teachers is a key to the integration of MOOCs and offline courses. Only when students accept MOOCs, can teachers’ teaching be meaningful (Yuan and Wu, 2020). MOOCs are powerful in classroom teaching and knowledge acquisition, and can broaden the breadth and depth of classroom teaching, effectively improving the teaching quality and learning efficiency. MOOCs change the traditional teaching concept and make students feel the new teaching method, which makes the traditional classroom less boring. The teaching mode in flip classrooms is a new form of MOOCs, and it inputs new power to classroom teaching. In this case, teachers should provide students with a foreign language atmosphere (Yang and Wu, 2019). This kind of reversal idea and mode is popular in foreign countries for a long time. Most foreign countries use MOOCs as teaching materials and achieve good results. MOOCs have become the mainstream teaching method. In China, many excellent educational institutions and colleges have built the MOOC platform and establish a wealth of knowledge resources’ networks, such as the C2 Alliance led by East China Normal University and the “1+1” platform established by the People’s Congress. Students can give full play to their expertise on this platform and show their advantages, which shows a feature of the flipped classroom (Zheng et al., 2018). In this way, students’ desire for autonomous learning is motivated. Direct communication and frequent interaction brought by MOOCs provide great conveniences for teachers and students. MOOCs can meet any students’ needs according to their aptitude and improves their learning efficiency, as shown in Figure 1.

### Collaborative Development of Massive Open Online Courses and English Grammar Teaching

In the teaching practice, the interlaced teaching method can benefit more. MOOCs have a wide range of knowledge, strong pertinence, and a high degree of freedom. Teachers can form their teaching style in MOOCs according to teaching experience and methods, as well as the specific teaching content, to arouse students’ interest, and encourage them to learn actively and independently. In the teaching process, teachers give students freedom in learning, so that everyone can express their ideas and give full play to the advantages of MOOCs in the communication between teachers and students. Under the heavy teaching tasks, teachers can learn from the micro-videos in MOOCs to let students understand the course content and outline the key points in advance (Zhang et al., 2018). This can help students learn from the traditional acceptance learning method to understand learning and happy learning. The integration of MOOCs and classroom teaching can make the learning process more interesting, improve the enthusiasm and happiness of learning, and overcome some learning barriers. For example, English syntax has finite and non-finite attributive clauses. Students often confuse the two clauses. MOOCs can give targeted explanations for the differences between the two clauses, and then match the content that can be practiced at any time, so that students can understand and master the differences as soon as possible, and overcome the fear of most students in the face of such grammatical points. In classroom teaching, some students do not take the initiative to ask questions. This phenomenon shows the limitations of traditional teaching methods. Some of the targeted courses of MOOCs are displayed in the form of micro-videos, and the difficulties and key points in the class are subdivided, like the difference between attributive clauses. After class, students can review them again, which can objectively improve students’ enthusiasm and learning effect.

**FIGURE 2:**
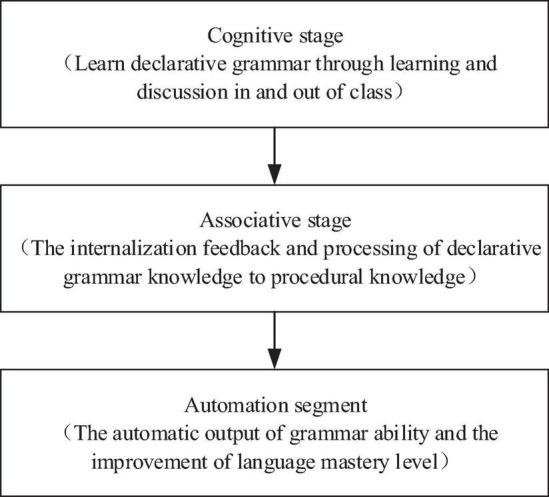
Basic knowledge acquisition of English grammar in a non-native language environment.

If they want to be proficient in using English grammar, students usually have to master the basic knowledge through many exercises, making the knowledge output correctly, as shown in Figure 2.

### Combination of Massive Open Online Course and English Grammar Teaching

Massive Open Online Courses about grammar learning extend the classroom teaching modes in traditional English grammar classrooms. They can deliver knowledge to students because they are the sources and backgrounds of the knowledge, which helps students fully understand the knowledge, arouse students’ curiosity, promote students’ learning motivation, develop their thinking, and master the knowledge. The long-term traditional classroom teaching is fixed and boring, and every student is learning for the college entrance examination, making students tired of study. MOOCs can relieve students’ mental health problems and make the learning process an active acceptance. Through extracurricular MOOCs, some APPs can make the learning process so relaxing that most of the students can learn knowledge happily. This teaching method integrating into life is also another great advantage that MOOCs bring. The integration of ordinary life and grammatical points makes the learning process interesting. Therefore, the efficiency of students’ language learning becomes better through short after-school micro-videos. For example, in the learning process of subject clauses and predicative clauses, some students cannot understand the differences between them. If the student still do not know about the differences between them from the videos, it requires students to communicate more with teachers on the MOOC platform and ask questions so that the questions can be understood correctly.

## Massive Open Online Course and Deep Learning

**FIGURE 3:**
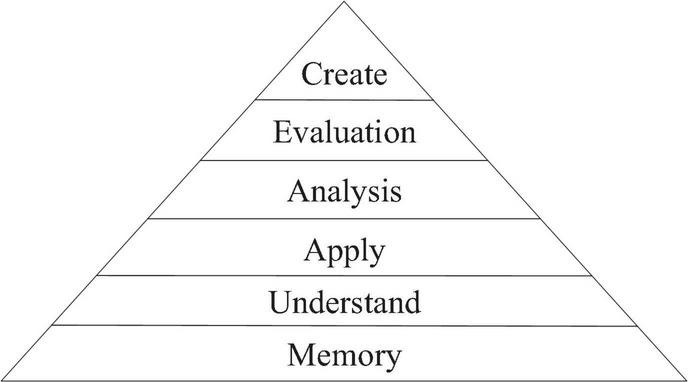
Bloom’s cognitive hierarchy.

Deep learning, as its name implies, deepens the understanding of learning content and critical thinking and have a profound impression on the knowledge. It can be skilfully applied to flexible movements in reality to solve complex problems. Opposite to deep learning, it is shallow learning, which only focuses on how to memorize input knowledge and its shallow understanding. Benjamin Bloom, an American educator, put forward his view in 1956. He argued that the goal of education is to complete the processes of cognition, emotion, and skill learning. In 2001, his student, WLorin Anderson, added his view to his teachers, suggesting that human cognition can be divided into creation, evaluation, analysis, application, understanding, and memory. Although Bloom’s view does not seem to have much to do with deep learning, the content and form of this theory are all consistent with the basic concept of deep learning as shown in Figure 3.

In 1976, Roger Saljo and Ference Marton jointly published the research on *Qualitative Differences in Learning: Outcome and Process*, in which deep learning and shallow learning are officially proposed for the first time. Thirty years later, American Association for Educational Communication and Technology (AECT) believes that deep learning can serve as a powerful boost to educational development. In China, Li Gahou also began to study deep learning after it is introduced in the United States. In 2008, Li Kedong mentioned the content of deep learning for the first time, which caused a sensation in the academic circles, and the related theoretical analysis appears from time to time. Connotation, theory, occurrence, teaching modes, and methods are the main contents in the study of deep learning in China.

First, the teaching mode of English grammar based on deep learning is a student-centered learning model. In this learning mode, students no longer passively memorize and recite language points summarized by teachers, but participate in discovering, mining, comparing, analyzing, summarizing, and applying the relevant knowledge themselves, making students have a thorough understanding of the language points and improve their ability to learn English grammar.

Second, the teaching mode of English grammar based on deep learning is a learning model that focuses on students’ thinking. In such a teaching mode, teachers should pay more attention to cultivate students’ thinking ability, including the analytical thinking ability, discriminative thinking ability, comparative thinking ability, and summative thinking ability. When students draw relevant conclusions through these thinking methods, teachers ask students to share their thinking within the group, and explain to peers their specific thinking process in the analysis, comparison, and synthesis, improving students’ thinking ability and communication ability.

### Deep Learning Platform Built by Massive Open Online Course for Students

**FIGURE 4:**
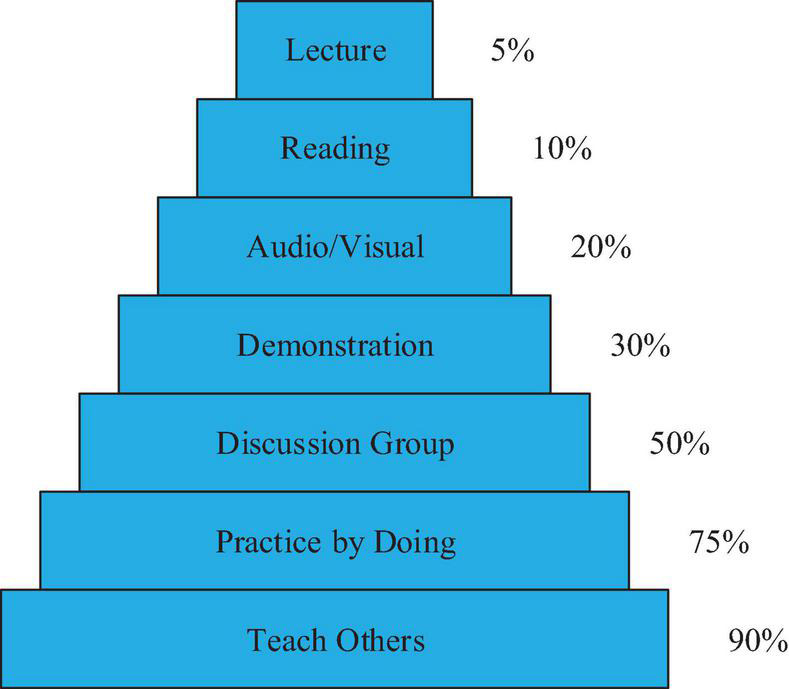
Knowledge acquired.

Massive Open Online Courses open a way of knowledge acquisition and maximize the public benefits of different classes, realize the knowledge transmission and information replication, and provide infant education (Litjens et al., 2017). No matter you have the relevant knowledge or not, you can make use of your spare time to study in MOOCs at any time, so that time and space are no longer resistance to learn knowledge. MOOCs have certain requirements for scholars, and they must have some cultural literacy. MOOC has a higher requirement for students, and it asks students to interact with teachers more effectively, especially in a class with fewer students. Teachers should be responsible and give more energy to teach students according to their aptitude so that each student can receive the most suitable learning method and take fewer detours of learning. Interaction directly improves students’ enthusiasm for study, and they are more willing to communicate with teachers. Effective interaction between students and teachers is a favorable condition for deep learning. In the MOOC classroom, students can learn the content in advance according to the micro-video before class, and realize the low-level learning methods such as memorizing and understanding learning methods by Bloom. Before class, the teacher should answer some of the questions raised, which can promote the smooth progress of the course and eliminate the doubts in advance. In classroom teaching, students can discuss and show their views with the help of teachers, making their cognitive abilities developed, such as application, judgment, and innovation. After the class, teachers can assign more difficult tasks and test students’ cooperative ability, improving their thinking and judgment skills. Students’ autonomous learning before the class and teachers’ targeted teaching after the class make learners’ satisfaction improved, as shown in Figure 4.

The deep learning teaching mode based on Small Private Online Course (SPOC) is the integration and innovation of MOOCs and the classroom learning mode, rather than the simple superposition of the two. The advantages are summarized in Table 2.

**TABLE 2:** Comparison of learning types between Massive Open Online Course (MOOC) and Small Private Online Course (SPOC).

Learning types	Target	Teaching modes
Shallow learning	Memorize	MOOC, SPOC online learning before class
	Understand	MOOC, SPOC online learning before class
Deep learning	Apply	SPOC in class and after class
	Analyze	SPOC in class and after class
	Evaluate	SPOC in class and after class
	Create	SPOC in class and after class

### Cultivating Students’ Ability to Apply, Evaluate, and Innovate New Knowledge by Massive Open Online Courses

First, the teachers’ identities should be transformed. The teacher is not only a knowledge importer but also a supervisor who monitors students’ autonomous learning based on deep learning. The general teaching cycle of MOOCs is about 1 month. Under this mode, the teacher needs to constantly guide and help students. This is the teaching content in the classroom, and the teaching design should be improved at any time after the class, and the students’ learning achievements should be paid more attention to. Supervision and answering questions should be what the teacher does before class. Through the examination questions and feedback questions on the micro-video, the teacher summarizes and selects meaningful questions and answers them in the classroom. In addition to solving the problem, teachers can also know about students’ attitudes and enthusiasm to study through MOOCs and have a certain understanding of the learning states of different students. In the face of difficult problems, the class can be organized to discuss them, and after-school tests can help students master knowledge better. Therefore, the combination of MOOCs and the traditional teaching mode truly realizes the student-centered and teacher-led teaching mode.

Due to the guidance of MOOCs, teachers’ teaching methods change a lot. Compared with the traditional teaching system, MOOCs often take only 1 to 2 months to complete a teaching task and save a lot of time. The rich knowledge base and the convenient platform provide a way to learn language points quickly. Small-scale MOOCs pay more attention to the personalized learning method. Before class, the teacher makes the personalized teaching courseware according to the requirements of each student, including key points and special points, and strives to take care of each student. Each student’s actual situation is not the same, and the easy question to the student may be difficult to others. Therefore, different students have different problems in learning a course, and a large number of problems increase the teachers’ challenge in the teaching process. That is, MOOCs bring many new challenges to teachers, and teachers should check students’ mastery of basic knowledge before class. In class, teachers should solve students’ common problems and focus on training the key points and difficulties. After class, teachers should have higher requirements for students and give them more difficult tasks. This undoubtedly increases the pressure on teachers, and they have to do more preparation.

### Deep Learning Mode of College English Teaching Based on Massive Open Online Courses

**FIGURE 5:**
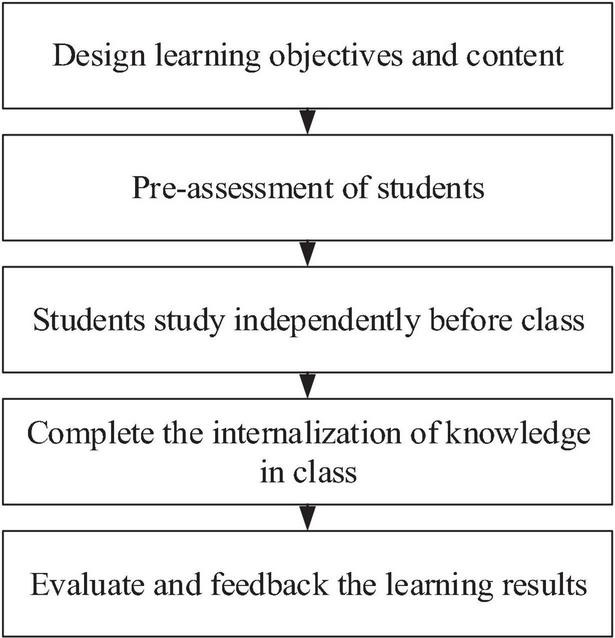
Deep learning mode of college English teaching based on Massive Open Online Courses (MOOCs).

The new deep learning is blended by online MOOCs and offline traditional courses. There are three teaching modes: online teaching before class, online teaching in class, and feedback after class, as shown in Figure 5.

The current situation of deep learning is constantly influencing traditional teaching methods and making them change greatly. The previous unilateral courses is a curriculum model, which involves frequent interaction, diligent cooperation, and research. High-quality online teaching resources help teachers make courseware and help students preview before the class, so that the learning process can be interesting, complete, and understandable. Online and offline teaching can make the knowledge digested better, solving the problems caused by online courses. At the same time, differentiated courses can meet different students, so that they have the ability of self-criticism and self-creation. Small tests after class can help students know about their problems and solve them in time. At present, English grammar courses in senior high schools is complex, the learning task is heavy, and its practicability is poor. In this case, the roll inspection is needed to help students learn English grammar well in healthy physical and mental states. And the fragmentation management of MOOCs provides a shortcut for students to learn and consolidate the knowledge in this way.

## Data Results Analysis

### Test Data

The test takes learners who participate in MOOCs as the subject to understand the effect of MOOCs based on deep learning. This test experiment has high requirements for information technology equipment, so Shaanxi Normal University and Bohai University with good digital teaching level and equipment are selected to carry out the teaching experiment. At present, all colleges and universities have a Gigabit optical fiber backbone network, and 200 Mbps export broadband is set to realize the wireless LAN coverage of the whole university. In addition, a multimedia information application development studio is established, and the world university town space is purchased and distributed for teachers and students freely.

English teacher Wang and Japanese teacher Li from Shaanxi Normal University and English teacher Tian and Japanese teacher Pan from Bohai University are selected to give lectures. These four teachers have rich teaching experience and are good at operating modern educational technology, meeting the relevant standards of the experiment. English majors and Japanese majors with similar situations are selected. Around 80% of English majors and 20% of Japanese majors are arranged in each class because these students have certain theoretical knowledge but lack practice. The number of experimental students in Shaanxi Normal University is 720 (640 in English and 80 in Japanese), and that of experimental students in Bohai College is 660 (600 in English and 60 in Japanese). In addition, as the students who participated in the experiment have similar learning interest, academic performance, and intelligent development, which met the research conditions, both universities have the basic conditions for conducting the experiment.

### Current Situation and Characteristics of College Students’ Cognition and Ability of Foreign Language Learning

Table 3 shows that the final scores of students who take MOOCs are much better than those who take regular courses. Because the students had learned the relevant knowledge before class, the discussion in class makes the difficult problems solved the fastest, and after class, they are tested. Deep learning significantly improves the teaching quality, and the average score of the students who take MOOCs based on deep is 89.6. The more striking thing is that the rate of students who score more than 85 increased from 15 to 75%. In the past 5 years, the average score of this rhetoric is about 65–70.

**TABLE 3:** Comparison of test scores between the traditional teaching mode and MOOC.

Before application	5	12	3
After application	1	4	15
Score	<60	60–85	85–100

**FIGURE 6:**
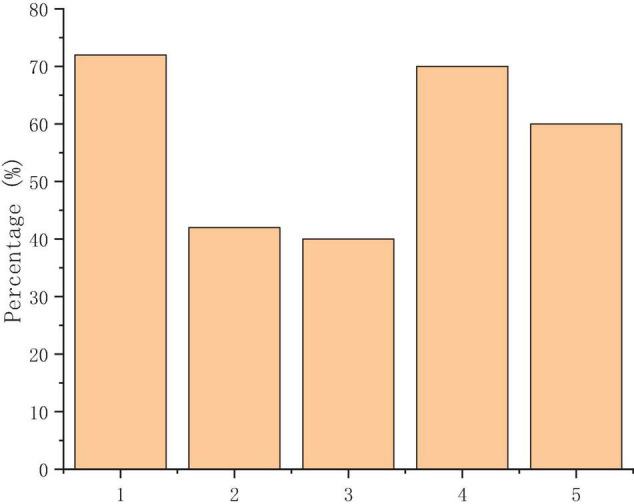
Main functions of the learning platform of MOOCs [(1) assistance in classroom learning; (2) the download of learning materials; (3) discussion and communication with peers; (4) autonomous learning; and (5) submitting homework and examination].

Figure 6 suggests that the MOOC platform is mainly used to assist students in classroom learning and developing their autonomous ability. However, fewer students choose to download learning materials and discuss with peers, which shows that students have no awareness of online learning and their enthusiasm for online interaction is low.

**FIGURE 7:**
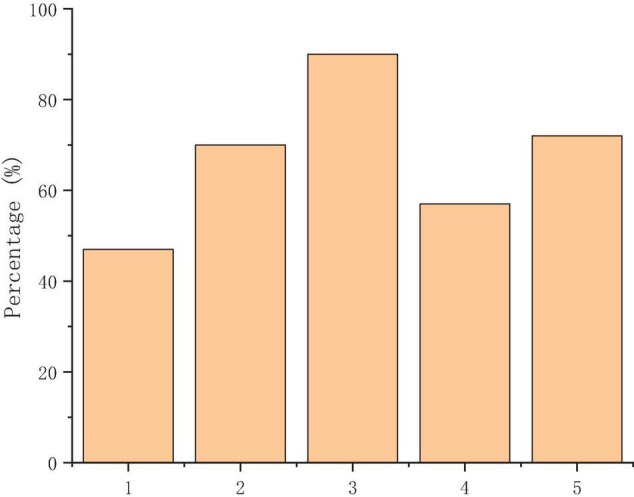
Achievements of the hybrid learning of MOOCs [(1) deepening the feelings with teachers and classmates; (2) improving autonomous learning ability; (3) expanding the knowledge; (4) improving the ability to solve problems; and (5) mastering the new way of MOOCs].

Figure 7 reveals that the students are generally satisfied with the learning effect of the hybrid learning of MOOCs. The students think that the hybrid learning of MOOCs is the most helpful to their practical operation skills, which shows that most students have mastered the methods and skills of micro class design through hybrid learning. Students generally believe that this learning expands their knowledge, improves their autonomous learning ability and problem-solving ability, and helps them to master a new way of MOOCs.

**FIGURE 8:**
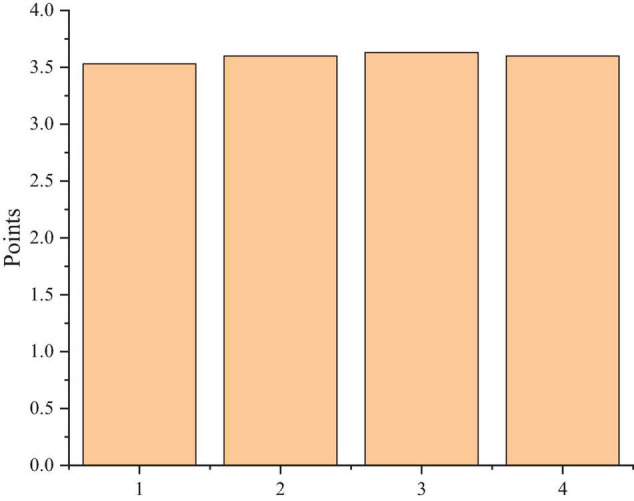
Satisfaction with the learning strategies of MOOCs.

In Figure 8, (1) is the help of peer discussion in learning; (2) is the help of mind map in learning; (3) is teachers’ help to students; and (4) is the help from assignments.

Figure 8 shows that students are satisfied with the mixed teaching strategy of MOOCs, and teachers play an extremely important role in the course. Teachers not only need to guide students to carry out autonomous learning of MOOCs, but also need to play the role of assistants online and offline, so that the whole learning becomes an online and offline interaction between teachers and students, as well as the communication between students.

College students are not familiar with the concept of deep learning, and they lack the awareness of English learning based on deep learning. Also, the results show that students’ sense of pleasure and satisfaction is low in English learning. They are unwilling to practice oral English and have no interest in grammar learning and practice. They think that college English learning is just for passing CET-4 and CET-6 instead of a useful tool for communication.

In the deep learning strategy, fewer students attend class or autonomously learn with questions, and most of them can ask the teacher their questions actively. For confused questions, most students spend their free time studying them, and only fewer students give up halfway.

### Construction of College Oral English and Speech Deep Teaching Model

The classic SPOC mode is the combination of MOOCs + the traditional classroom mode. It includes three stages such as before class, in class, and after class. The before class stage is mainly for teachers to design and develop teaching resources, and students independently practice phonetics, tones, stress, and reading on the online platform, previewing knowledge in advance, preparing questions, and conducting online communication and practice. The activities of in-class stage are that teachers communicate with students, conduct classroom discussion, evaluate learning effect, correct students’ pronunciation and intonation errors, answer questions, eliminate doubts, and internalize knowledge. After class, teachers and students conduct online practice according to their learning needs or choose offline communication and practice. The main content is language use practice. Students jointly complete group tasks through online group cooperation, realizing knowledge sublimation and completing the deep learning.

**FIGURE 9:**
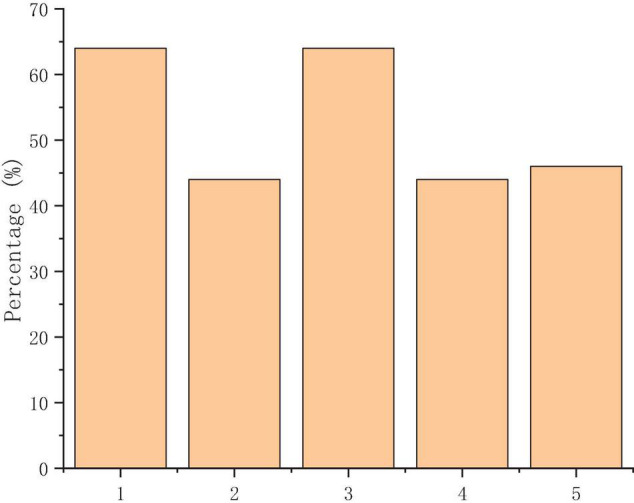
Factors affecting hybrid learning [(1) a weak correlation between network resources and courses; (2) unavailable to online learning; (3) insufficient time; (4) heavy tasks assigned by teachers; and (5) inconvenient to use the Internet].

Figure 9 reflects that many students believe that time and Internet access also affect hybrid learning of English. Few students can access to online learning and their learning enthusiasm is lowered. Some students also complain that teachers assign too many tasks, which reduce their interest in learning. It required the continuous guidance of instructors, providing students with convenient Internet access conditions as much as possible and helping learners integrate into the learning environment to stimulate their learning motivation.

### Learning Effectiveness Evaluation

The comparison of the test data indicates that the scores of students in the classic teaching mode are higher than those in the online mode, and the number of students in the classic teaching mode scoring 80–90 points is higher than that in the online mode.

In the first stage, the experimental results are shown in Table 4. In the second stage, the following information is obtained through interviews. The fully online mode saves a lot of time and has great time flexibility. However, the advantages of the classic mode are also obvious. First, face-to-face classroom teaching is adopted in the classic mode, so students feel that the supervision mechanism is stronger than machine statistics. Although students in the fully online mode can communicate with teachers online, many students’ online performance is poor. This indicates that the supervision mechanism is weak, and students’ initiative and self-control ability should be improved. Second, due to the particularity of oral courses, the face-to-face teaching mode can more clearly show the pronunciation and mouth shape of students and teachers, which is more conducive to the practice of oral pronunciation. Third, due to the instability of the network and equipment, the interaction efficiency is low between online teachers and students, resulting in a waste of time in class. In addition, this course also needs speech competitions, and offline face-to-face guidance has a greater advantage, which alleviates teachers’ guidance on students and their expressions. Therefore, face-to-face practice is closer to the competition scene and better than online guidance. In the third stage, although students can leave messages and ask questions to teachers on social software or the network platform, the answers to these questions will be delayed or ignored. Therefore, offline face-to-face question–answering is timely and efficiently for students. In short, students can use their fragmented time to carry out MOOCs based on deep learning. In the process of online learning, teachers and students can effectively interact and realize the teaching objectives. The mixture of the online and offline teaching modes is a successful practice, which should be widely recognized.

**TABLE 4:** Experimental results of two modes.

Results	Classic mode	Fully online mode
Progress rate	50%	75%

## Conclusion

The application of MOOCs based on deep learning to college students’ English grammar teaching is studied. Through data investigation, it is found that the multimedia resources of the teaching mode based on deep learning present rich knowledge in the forms of texts, animations, sounds, or films and TVs, which are embedded with problems, tasks, and tests, promoting students’ active knowledge construction and in-depth understanding of new knowledge. Students can watch the teaching resources on the MOOC platform anytime and anywhere to review them autonomously. The students who are left behind can get timely guidance from teachers in the early stage of new knowledge teaching so that they do not be left behind in the later period. High-quality online teaching resources and the teaching environment based on deep learning can provide a variety of interactive tools, by which students can communicate with their peers and teachers online, and discuss questions and acquire knowledge. Sharing open online communication, classroom discussion, and situational simulation can enhance students’ deep learning abilities such as their abilities of language communication and transfer application. Constructivism with interaction as its core achieves a grasp of new knowledge. Extensive communication and interaction are important ways for deep learning, thinking, and criticism. The new model emphasizes giving students a deep learning experience and developing the teaching resources of MOOCs that are popular all over the world, making the knowledge rooted on the campus, realizing the combination of online resources and campus classroom teaching, and maximizing the interaction between online and offline teachers and students. Students can acquire knowledge through autonomous learning and discussion between teachers and students before class, which is helpful to ensure deep learning. For in class and after class, the internalization and sublimation of knowledge are completed by means of group collaboration, inquiry learning, situational simulation, and evaluation, which drives students to construct new knowledge actively, highlighting the dominant position of students.

In practice, the deep learning model does not promote students’ deep learning to a large extent, which attributes to the improper use of the method, especially teachers’ insufficient awareness and knowledge reserves. For example, the 3rd-year English majors of Northwest A&F University have no enough time for autonomous learning before class, and they have to take classes from Monday to Friday. Also, the heavy academic burden makes students reluctant to ask questions and participate in forum exchanges actively if teachers still require students to complete pre-class preview. The impact of MOOCs on physical courses in colleges and universities is still an urgent problem to be solved. Teachers need to completely change their concepts, integrate various online resources before class, and maintain teaching enthusiasm and vitality in class. This puts new demands on teachers to master modern technology and professional knowledge. In summary, the transformation of teachers’ ideas and concepts is a basis for deep learning. Also, MOOCs require teachers to regard teaching as a lifelong process, make a reasonable teaching plan, and explore an efficient teaching method and strategy.

The research shows that MOOCs based on deep teaching can evoke college students’ enthusiasm and participation in English learning. This has a positive effect on students’ mastery of basic knowledge, acquisition of practical skills, and teamwork ability. With the continuous development of information technology, the Internet and personal mobile devices are becoming more and more popular, and the learning at the fragmented moment is advocated. MOOCs based on deep learning should be explored further, and the next research work should be carried out from the following aspects:

(1) Research on MOOCs for different subjects. In the study of MOOCs based on deep learning, only some chapters of the book “English Grammar and Curriculum Integration” are selected, and the conclusions may not apply to other types of courses. It needs to be discussed whether there are differences in the implementation of MOOCs in different types of courses. Therefore, MOOCs based on deep learning should be practiced in different types of courses, and the research scope should be expanded.
(2) Big data learning analysis based on the MOOC platform. The results show that students’ online learning enthusiasm is not high without monitoring, and teachers cannot record, understand, and analyze students’ learning behavior through big data on the MOOC platform. The big data analysis on the MOOC platform is to track and record students’ learning behavior comprehensively and systematically. Teachers can know about and analyze their learning preferences, curriculum completion rate, participation and interaction, and course selection to carry out individualized teaching for different students. In future, big data learning analysis on the MOOC platform should be emphasized, and personalized learning programs should be made according to students’ learning behaviors.
(3) Construction of the localized MOOC resources. In the application research of MOOCs based on deep learning to college students’ English grammar, it is found that there are many high-quality and rich MOOC resources on the Internet. It is easy to ignore the particularity of students and courses in the university by simply relying on the introduction of famous teachers. In the construction of MOOC resources, colleges and universities should fully consider the characteristics of local network courses, by which high-quality MOOC resources should be reorganized. This will be one of the main tasks of subsequent research.

## Data Availability Statement

The raw data supporting the conclusions of this article will be made available by the authors, without undue reservation.

## Ethics Statement

The studies involving human participants were reviewed and approved by the Changshu Institute of Technology Ethics Committee. The patients/participants provided their written informed consent to participate in this study. Written informed consent was obtained from the individual(s) for the publication of any potentially identifiable images or data included in this article.

## Author Contributions

Both authors listed have made a substantial, direct, and intellectual contribution to the work, and approved it for publication.

## Conflict of Interest

The authors declare that the research was conducted in the absence of any commercial or financial relationships that could be construed as a potential conflict of interest.

## Publisher’s Note

All claims expressed in this article are solely those of the authors and do not necessarily represent those of their affiliated organizations, or those of the publisher, the editors and the reviewers. Any product that may be evaluated in this article, or claim that may be made by its manufacturer, is not guaranteed or endorsed by the publisher.
